# In Vitro Cultures of Some Medicinal Plant Species (*Cistus* × *incanus*, *Verbena officinalis*, *Scutellaria lateriflora*, and *Scutellaria baicalensis*) as a Rich Potential Source of Antioxidants—Evaluation by CUPRAC and QUENCHER-CUPRAC Assays

**DOI:** 10.3390/plants10030454

**Published:** 2021-02-27

**Authors:** Michał Dziurka, Paweł Kubica, Inga Kwiecień, Jolanta Biesaga-Kościelniak, Halina Ekiert, Shaimaa A. M. Abdelmohsen, Fatemah F. Al-Harbi, Diaa O. El-Ansary, Hosam O. Elansary, Agnieszka Szopa

**Affiliations:** 1 The Franciszek Górski Institute of Plant Physiology, Polish Academy of Sciences, ul. Niezapominajek 21, 30-239 Kraków, Poland; m.dziurka@ifr-pan.edu.pl (M.D.); j.koscielniak@ifr-pan.edu.pl (J.B.-K.); 2 Chair and Department of Pharmaceutical Botany, Medical College, Jagiellonian University, Medyczna 9, 30-688 Kraków, Poland; p.kubica@uj.edu.pl (P.K.); inga.kwiecien@uj.edu.pl (I.K.); halina.ekiert@uj.edu.pl (H.E.); 3 Physics Department, Faculty of Science, Princess Nourah Bint Abdulrahman University, Saudi Arabia, Riyadh 84428, Saudi Arabia; shamohamed@pnu.edu.sa (S.A.M.A.); alharbiffa@gmail.com (F.F.A.-H.); 4 Precision Agriculture Laboratory, Department of Pomology, Faculty of Agriculture (El-Shatby), Alexandria University, Alexandria 21545, Egypt; diaa.elansary@alexu.edu.eg; 5 Plant Production Department, College of Food and Agricultural Sciences, King Saud University, P.O. Box 2455, Riyadh 11451, Saudi Arabia; helansary@ksu.edu.sa; 6 Floriculture, Ornamental Horticulture, and Garden Design Department, Faculty of Agriculture (El-Shatby), Alexandria University, Alexandria 21545, Egypt; 7 Department of Geography, Environmental Management, and Energy Studies, University of Johannesburg, APK Campus, Johannesburg 2006, South Africa

**Keywords:** antioxidant activity, plant in vitro cultures, CUPRAC and QUENCHER-CUPRAC assays, soil-grown plants

## Abstract

Comparative estimations of the antioxidant activity of methanolic extracts from biomasses of different types of in vitro cultures of *Cistus × incanus, Verbena officinalis, Scutellaria lateriflora*, and *S. baicalensis* and also from plant raw materials were performed. The antioxidant measurements were based on the modern assays—cupric ion reducing antioxidant capacity (CUPRAC) and quick, easy, new, cheap, and reproducible CUPRAC (QUENCHER-CUPRAC). The total extractable antioxidants (CUPRAC assay) ranged from 10.4 to 49.7 mmol (100 g)^−1^ of dry weight (DW) expressed as Trolox equivalent antioxidant capacity (TEAC), and the global antioxidant response (QUENCHER-CUPRAC assay) ranged from 16.0 to 79.1 mmol (100 g)^−1^ DW for in vitro cultures, whereas for plant raw materials the total extractable antioxidants ranged from 20.9 to 69.5 mmol (100 g)^−1^ DW, and the global antioxidant response ranged from 67.2 to 97.8 mmol (100 g)^−1^ DW. Finally, the in vitro cultures could be regarded as an antioxidant-rich alternative resource for the pharmaceutical, health food and cosmetics industries.

## 1. Introduction

Oxidation processes are inherent for the energy generation to sustain biological activity in living organisms. Hence, the unrestrained production of oxygen reactive species (ROS) is entangled in the origination of many chronic diseases, i.e. cancer, rheumatoid disease, atherosclerosis, and the degenerative processes associated with senescence [1,2,3,4,5]. To limit ROS damage, synthetic and semisynthetic antioxidants are extensively used [2,6,7] but, they have been suspected to bear responsibility for cell or whole-organ (liver) injuries and carcinogenesis [8,9]. Thus, there is a great need for functional and natural antioxidants that are able to reduce ROS overproduction and slow down the advancement of many chronic diseases. Plant-derived natural antioxidants are very efficient at inhibiting the process of oxidation by neutralizing ROS. Additionally medications derived from plant sources are considered safer than synthetics [10].

The total antioxidant capacity (TAC) could be considered as an effective marker for assessing plant material value in the context of antioxidants. One of the most effective, recent methods of TAC estimation is the CUPRAC (cupric ion reducing antioxidant capacity) assay [11]. This method possesses multiple advantages, including the fact that measurements are done at a neutral pH (about 7), which is more representative of living systems. The results are additive, i.e., TAC for phenolic mixtures is about equivalent to the sum of the antioxidative capacities of the individual ingredients [12]. The modification of the CUPRAC method is QUENCHER-CUPRAC method (quick, easy, new, cheap, and reproducible treatment, involving forced solubilization of bound phenolics by oxidizing TAC reagent). This approach allows for the inclusion of interactions at the edge between the solid phase (sample matrix antioxidants trapped within) and liquid phase (containing soluble oxidants (e.g., ROS), indicators, or probes) to estimated TAC [5,6,13].

The study aimed to evaluate selected in vitro cultures of important medicinal plants in correspondence with soil-grown plant material. The important context of the work is the comparison of antioxidative capacity and the accumulation of secondary metabolites characteristic of the evaluated plant species. *Cistus × incanus* L., the pink rock-rose (hairy rockrose), is important in the traditional medicine. The raw plant material contains exceptional amounts of polyphenols, mainly flavonoids, tannins, proanthocyanidins, and gallic acid, responsible for the strong antioxidant potential and anti-inflammatory, anti-rheumatic, anti-ulcer, anti-microbial, and immunostimulatory properties [14,15]. The *Verbena officinalis* L., vervain, is a medicinal herb broadly distributed in the world. Plant raw material can protect cells and tissues from oxidative injuries and stimulate physiological resistance [16]. The main constituents are phenylethanoid glycosides (verbascoside and isoverbascoside), iridoid glycosides (verbenalin and verbenin), and phenolic acids [17,18]. *Scutellaria baicalensis* Georgi, Baikal skullcap, is typical of Eastern Asia [19,20]. *Scutellaria lateriflora* L. is a known medicinal plant of North America. Both skullcaps show strong antioxidant and other valuable properties, i.e., antibacterial, antiviral, antifungal, antiallergic, antioxidant, anti-inflammatory, anticoagulant, anticancer, hepatoprotective, cholagogic, and sedative [21]. Plant material contains high amounts of phenolic compounds, such as the specific flavonoids (baicalin, baicalein, wogonoside, wogonin, and scutellarein), and also phenylethanoid glycoside (verbascoside), iridoid glycosides, phenolic acids, and tannins [21].

In this study, extracts from the plant material from in vitro cultures of *C. × incanus, V. officinalis, S. lateriflora,* and *S. baicalensis,* as well as from plant raw materials of soil-grown plants, were analyzed. Biomass from in vitro cultures can produce high quantities of secondary metabolites with valuable biological activities, e.g., different groups of polyphenols with antioxidant properties [10]. Recently, our results of biotechnological studies of medicinal plant species proved this to be the case, based on previous research on the plant metabolites with antioxidant activity, accumulating in in vitro cultures of the chosen plants [15,17,22]. It is noteworthy that the biosynthesis of metabolites in in vitro cultures could be easily controlled and stimulated. In plants growing under open field conditions, great differences in biosynthetic potential and consequently in therapeutic value have been documented. Moreover it is possible to establish and maintain the in vitro cultures of precious or even endangered plants from all climate zones of the world [15,23].

## 2. Results and Discussion

For in vitro-cultured biomass, the total extractable antioxidants (detected by the CUPRAC assay) ranged from 10.4 to 49.7 mmol (100 g)^-1^ DW for *S. lateriflora* and *V. officinalis*, respectively. The global antioxidant response (QUENCHER-CUPRAC assay) ranged from 16.0 to 79.1 mmol (100 g)^−1^ DW *(S. lateriflora* and *C. × incanus,* respectively). For plant raw materials, the total extractable antioxidants ranged from 20.9 to 69.5 mmol (100 g)^−1^ DW (*V. officinalis* and *C. × incanus,* respectively). The global antioxidant response ranged from 67.2 to 97.8 mmol (100 g)^-1^ DW for *S. baicalensis* and *V. officinalis,* respectively (Table 1).

Antioxidant capacity is strongly related to primary and secondary metabolism which are of the greatest interest to pharmacy. Typical plant secondary metabolites that have antioxidant potential are considered to be mainly polyphenolic compounds. For this reason, we estimated quantities of selected groups of plant polyphenolic compounds, i.e., phenolic acids, phenylethanoid glycosides, catechins, and flavonoids. The data acquired from targeted profiling of the analyzed plant extracts are collected in Table 2. Significant differences were found between extracts from plant material grown in different in vitro culture systems (stationary and agitated). Differences were also noted between soil-grown plant raw materials.

Methanolic extracts from biomass from in vitro *C.* × *incanus* agitated shoot cultures showed high TEAC response (Table 1A). Noteworthy are the results of the global antioxidant response of agitated biomass (79.1 mmol (100 g)^−1^ DW), which exceeded those of soil-grown plant material (72.0 mmol (100 g)^−1^ DW). The global antioxidant response of the material grown on agar was 30% lower. Those dependencies were well confirmed by the results for the selected groups of polyphenols, particularly phenolic acids, catechins, and flavonoids (Table 2A).

The results of *V. officinalis* showed that the global antioxidant response of agar cultures (64.0 mmol (100 g)^−1^ DW) was two times higher as for agitated cultures (32.1 mmol (100 g)^−1^ DW) (Table 1B). The total extractable antioxidant contents of both tested in vitro cultures were also more than two-fold higher than for soil-grown raw material (20.9 mmol (100 g)^−1^ DW) (Table 1B). Also, the accumulation of the selected groups of polyphenolic compounds, especially of phenylethanoid glycosides, were higher for in-vitro-cultured biomass than for soil-grown material (Table 2B).

Extracts from in vitro biomass of *S. baicalensis* showed higher TEAC values than the in vitro biomass of *S. lateriflora*; total extractable antioxidants—13.3 and 10.4 mmol (100 g)^−1^ DW, and global antioxidant response—26.7 and 16.0 mmol (100 g)^−1^ DW, respectively (Table 1C). Extracts from the plant raw materials analyzed under the current study by the CUPRAC and QUENCHER-CUPRAC methods behaved similarly; the total extractable antioxidants were about 32–33 mmol (100 g)^−1^ DW, and the global antioxidant response, about 67–69 mmol (100 g)^−1^ DW (Table 1C). The high antioxidant potential of both skullcaps was proved by the high contents of flavonoids characteristic to the *Scutellaria* genus in the plant raw material (Table 2C).

Analyzing Table 1, an interesting phenomenon can be observed—the antioxidant potential of methanolic extract tends to be lower than global antioxidant response assayed by the QUENCHER method. In this case, an explanation could be the background of this assay. The signal is also generated by insoluble antioxidants or antioxidants captured in insoluble matrix clusters [6]. Further, it can be seen that the vegetation conditions, soil, or in vitro conditions introduce variation among antioxidant status. The reason for that data could be that plant secondary metabolism is very complicated and the mutual balance of the metabolites could be connected to the reaction of the plant to environmental stimulations. Highly controlled conditions of in vitro culture are still prone to even slight variability, which could lead to different plant responses. Further, plants grown in field conditions are exposed to a virtually uncontrolled environment. In this case, huge variations between vegetation seasons could be observed. This is mirrored in plant physiology and secondary metabolism, and thus the balance of particular compound groups, and active substances.

**Figure 1:**
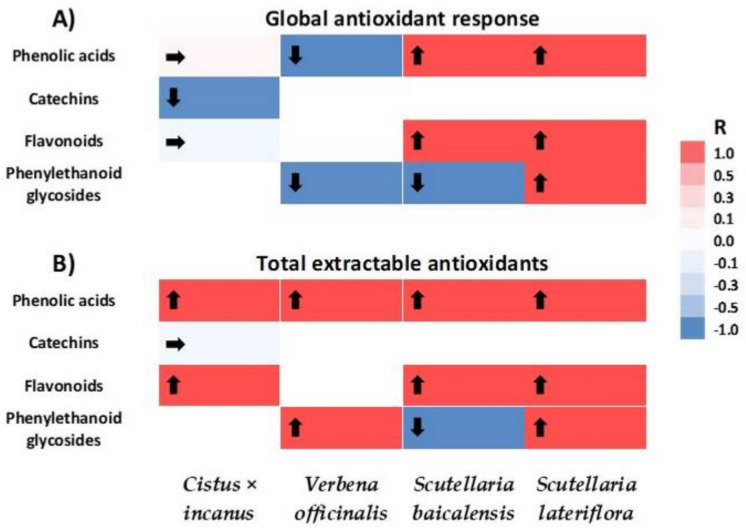
Heat map of correlation between phenolic acids, catechins, flavonoids, and phenylethanoid glycosides and antioxidant potential in biomasses cultured in vitro and in soil-grown plant raw materials of *Cistus* × *incanus***,** *Verbena officinalis,* and *Scutellaria baicalensis,* and *Scutellaria lateriflora*. Global antioxidant response panel (**A**), and total extractable antioxidants panel (**B**). Color saturation represents the intensity, whereas arrows represent the direction of correlation coefficient value (R) change.

In this work, we have presented data on antioxidative properties and the accumulation of important secondary metabolites of selected medicinal plants. Figure 1 represents a heat map of the relationship between those metabolites and antioxidative potential regardless of growing conditions. It can be seen that antioxidant response depends not only on species but also the compound group. All estimated metabolites can be linked to a wide group of phenolic compounds. This is a big fraction of secondary metabolites, broadly present in plants. Considerable variation has been reported in phenolic compounds of diverse species, which was also noticed in our study. Since there is complexity and variability of the natural mixtures of phenolic compounds in a vast number of plant preparations, it is relatively hard to illustrate each compound and clarify its structure, it is not problematic to identify main groups of significant phenolics. Numerous medicinal plants have been investigated and their phenolic composition is recognized to some extent [10]. The amounts of phenolic compounds, as estimated by the chromatographic methods, vary from the values reported using the spectrophotometric method (i.e., Folin–Ciocalteu (FC) reagent method). Further, the quantity of polyphenols is also reliant on the extraction methodology. Reports by many authors have also indicated that aglycones show higher antioxidative potential than glycosides [24].

The in vitro cultured biomasses/tissues could be regarded as more than satisfactory, and being even richer in antioxidants, an alternative source of valuable plant material [10]. Biomass from in vitro cultures can produce a higher yield of phenolic compounds, which means that in terms of antioxidants it is more valuable. This had been demonstrated for example by the comparative studies of callus tissue and soil-grown plant leaf extracts of *Stevia rebaudiana*, which were tested for their total phenolics according to the general/simple FC assay, and for the total antioxidant potential by FRAP and DPPH assays. The highest scavenging of the DPPH radical in the tested extracts was observed for methanolic extracts from callus cultures [25]. Also, Krolicka et al. [26] showed that some flavonoids are responsible for the high antioxidant potential of in vitro cultures of *Drosera aliciae*, detected with FRAP and DPPH tests. Moreover, the in-vitro-cultured biomass of *Ormenis africana* showed higher antioxidant power expressed in ABTS and DPPH tests than plant raw material [27]. The study conducted by Kovacheva et al. [28] assessed the radical scavenging activity (RSA) of a *Lavandula vera* MM cell culture extracts characterized by diverse rosmarinic acid accumulation. The authors compared the results with standard caffeic and rosmarinic acids solutions. The methods used were superoxide anion ABTS radical scavenging assays. Extracts from *Lavandula vera* MM cell lines possessed high RSA; the highest activity showed the fractions with enriched rosmarinic acid content [28]. On the other hand, comparative studies on different types of shoot cultures of *Artemisia judaica* (stationary liquid, agitated, agar, and bioreactor cultures) and in vivo plant material showed significantly higher antioxidant activity, based on the DPPH scavenging assay of extracts of mature greenhouse-grown plants [29]. Furthermore, Sökmen et al. [30] showed that extracts of herbal parts of *Origanum acutidens* exhibited a slightly better antioxidative potential than extracts of callus cultures. Notwithstanding the above, Grzegorczyk et al. [31] claimed that there were no significant differences between *Salvia officinalis* in vitro cultures and planted in vivo shoots in terms of antioxidant activity estimated by the DPPH method.

All of the studies based on the estimation of antioxidant potential of biomass from in vitro cultures have been based on simple, colorimetric assays like DPPH, FRAP, or superoxide radical scavenging [10]. The CUPRAC assay and its complete amplification—the QUENCHER-CUPRAC assay—are the newest, most effective and cheapest methods of measuring antioxidant potential [13]. The main strengths of these methods are the small amounts (in milligrams) of the required biomass samples and volumes of reagents, the neutral chemical environment of reaction (pH = 7), low cost, and easy reproducibility of all procedures. The results obtained by us prove the high importance of biotechnological studies for the pharmaceutical, health-food, and cosmetics industries. In in-vitro-cultured biomass, it is possible to manipulate the biosynthesis and accumulation of valuable metabolites, including different clusters of polyphenols with high antioxidant activity.

## 3. Materials and Methods

### 3.1. In Vitro Cultures

In vitro cultures of: *Cistus × incanus* (Cistaceae), *Verbena officinalis* (Verbencaceae), *Scutellaria baicalensis* (Lamiaceae) and *Scutellaria lateriflora* (Lamiaceae), were studied. The cultures were established in the Department of Pharmaceutical Botany, Jagiellonian University, Medical College (Kraków, Poland), for details see, respectively [15,17,22].

The in vitro cultures of the studied plants were cultivated under 4-week growth cycles on the Murashige and Skoog standard medium (MS, #M9274, Sigma-Aldrich, Poznań, Poland) (1962) with 3% (*w/v*) sucrose and growth regulators—*C.* × *incanus* (microshoot culture) with 3 mg L^−1^ BA (6-benzyladenine, #B3408, Sigma-Aldrich, Poznań, Poland) and 1 mg L^−1^ NAA (1-naphthaleneacetic acid, #N0640, Sigma-Aldrich, Poznań, Poland); *V. officinalis* (callus culture) with 1 mg L^−1^ BA and 1 mg L^−1^ IBA (indole-3-butyric acid, #I5386, Sigma-Aldrich, Poznań, Poland); and both *Scutellaria* (microshoot cultures) with 1 mg L^−1^ BA and 0.5 mg L^−1^ NAA.

The medium for all the tested plants was solidified with 7.2 g agar/L (*w/v*) (#P1001, Duchefa Biochemie, Haarlem, Netherlands), at pH 5.8 adjusted prior to autoclaving (20 min at 121 °C). Erlenmeyer flasks were used to keep the cultures.

In vitro cultures of *C. × incanus* and *V. officinalis*, were additionally conducted in agitated systems as microshoots and suspension cultures, respectively (Altel rotary shaker, 140 rpm, 35 mm vibration amplitude). Agitated cultures were maintained in 100 mL medium in Erlenmeyer flasks (inoculum, 1 g callus of *V. officinalis*, or 2 g microshoots of *C. × incanus*).

Cultures were cultivated in a plant growth room at 25 ± 2 °C under constant fluorescent white light of 4 W m^−2^ (LF-40 W, Pila, Poland).

### 3.2. Soil-Grown Plants Raw Materials

The plant material consisted of air-dried herbs (overground parts of plants with flowers) of *C. × incanus*, *V. officinalis*, and *S. lateriflora*, and air-dried roots of *S. baicalensis*. *C. × incanus,* and *V. officinalis,* which were collected in the Garden of Medicinal Plants of the Faculty of Pharmacy, Jagiellonian University, Medical College (Kraków, Poland). They were harvested in August 2017 in their mature vegetative growth phase. *S. baicalensis* and *S. lateriflora* came from imports from China and North America, respectively (Nanga, Zamkowa 97 Street, Złotów, Poland).

### 3.3. Total Extractable Antioxidants

The CUPRAC method [32] was slightly modified by Biesaga-Kościelniak et al. [33]. Lyophilized materials were ground to a uniform powder. Antioxidants were extracted with 1 mL of methanol (#32213-M, Sigma-Aldrich, Poznań, Poland) from 5 mg samples (15 min, 30 Hz; MM400, Retch, Haan, Germany). Samples were centrifuged (5 min. at 22,000 × g, UniversalR32, Hettich, Tuttlingen, Germany), 50 µL of the supernatant was pipetted to wells filled 50 µL of 10 mmol L^−1^ Cu^2+^ (#307483, Sigma-Aldrich, Poznań, Poland), 7.5 mM neocuprine (#N1501, Sigma-Aldrich, Poznań, Poland), and 1 mol L^−1^ (pH 7.0) ammonia-acetate (#238074, Sigma-Aldrich, Poznań, Poland) buffer. After 15 min incubation at 25 °C absorbance at 425 nm was recorded (Synergy 2, Winooski, VT, USA). The content of antioxidants was calculated as Trolox equivalent antioxidant capacity (TEAC) in mmol (100 g)^−1^ of dry weight (DW).

### 3.4. Global Antioxidant Response

The global antioxidant response was assayed employing QUENCHER-CUPRAC [13] altered to microwell plate formats. Ten mmol L^−1^ Cu^2+^, 7.5 mM neocuprine, and 1 mol L^−1^ pH 7.0 ammonia-acetate buffer and methanol were dispensed (1 mL) to a test tube with a 1 mg sample. After 30 min. of shaking, all samples were centrifuged and pipetted to 96-well plates. Absorbance was measured at 425 nm (Synergy 2). The antioxidant response was presented as TEAC in mmol (100 g)^−1^ of dry weight (DW).

### 3.5. Targeted Profiling of Natural Biologically Active Phenolic Compounds

Dried, pulverized plant material (described above), was extracted with methanol (50 mL per 0.5 g of sample) for 2 h under reflux. In the extract, the content of free phenolic acids, catechins, flavonoids, and phenylethanoid glycosides was analyzed. Quantification was performed using a modified HPLC method. All procedures were described earlier [15,17,22,34].

### 3.6. Statistical Analysis

The measurements were done in five replicates. The data were presented as mean with standard deviation (SD). Data were compared with the Wilks’ lambda test in multivariate analysis of variance (MANOVA) using STATISTICA 12 PL (StatSoft, Kraków, Poland) in collaboration with Princess Nourah bint Abdulrahman University and King Saud University.

## 4. Conclusions

In the study, comparative estimations of the antioxidant activity of extracts from biomasses of different types of in vitro cultures of four medicinal plant species, *C.* × *incanus*, *V. officinalis*, *S. lateriflora*, and *S. baicalensis* and also from plant raw materials of soil grown plants were done. The selected plants are of great interest because of their wide pharmacological potential. The work show that a plant’s culture conditions have a huge impact on its secondary metabolism. Antioxidant properties of the plant material should be assayed not only as a simple extract but should also involve forced solubilization of bound antioxidants.

Based on our estimations, we claim that the in vitro cultures of these plants could be regarded as plausible, an antioxidant-rich alternative origin of valuable raw material for the pharmaceutical, health food, and/or cosmetics industries in which the production could be controlled and stimulated.

## Figures and Tables

**Table 1:** Trolox equivalent antioxidant capacity (TEAC (mmol (100 g)^−1^ dry weight (DW)) in extracts of biomasses from in vitro and soil-grown plant raw materials of *Cistus* × *incanus* **(A)**, *Verbena officinalis* **(B)**, and *Scutellaria baicalensis* and *Scutellaria lateriflora* **(C)**. Mean values ± SE, the same superscript letter means lack of statistical significance between treatments (n = 5, *p* < 0.05).

**Antioxidant Response**	**TEAC (mmol (100 g)^−1^ DW)**					
	**Stationary Culture**		**Agitated Culture**		**Soil-Grown Plant Raw Material (Herb)**	
**(A)** ***Cistus* × *incanus***						
Total extractable antioxidants (methanol)	35.1a ± 1.2		42.3b ± 2.4		69.5c ± 3.2	
Global antioxidant response	48.6a ± 3.2		79.1b ± 2.2		72.0b ± 12.2	
**(B)** ***Verbena officinalis***						
Total extractable antioxidants (methanol)	44.0b ± 2.1		49.7c ± 3.4		20.9a ± 2.8	
Global antioxidant response	64.0b ± 2.3		32.1a ± 5.2		97.8c ± 4.5	
**(C)** ***Scutellaria baicalensis* and *Scutellaria lateriflora***						
**Antioxidant Response**	**TEAC (mmol (100 g)^−1^ DW)**					
	***Scutellaria baicalensis***			***Scutellaria lateriflora***			
	**Stationary Culture**	**Soil-Grown Plant Raw Material (Root)**		**Stationary Culture**		**Soil-Grown Plant Raw Material (Herb)**	
Total extractable antioxidants (methanol)	13.3a ± 4.5	33.1b ± 6.5		10.4a ± 2.5		31.5b ± 3.5	
Global antioxidant response	26.7b ± 4.5	67.2c ± 8.5		16.0a ± 4.5		68.7c ± 13.5	

**Table 2:** Contents of different subgroups of polyphenolic compounds detected earlier by us (mmol (100 g)^−1^ DW) in biomasses cultured in vitro and in soil-grown plant raw materials of *Cistus* × *incanus* acc. [15] **(A)**, *Verbena officinalis* acc. [17] **(B)**, and *Scutellaria baicalensis* and *Scutellaria lateriflora* acc. [22] **(C)**. Mean values ± SE, the same superscript letter means lack of statistical significance between treatments (n = 5, *p* < 0.05), tr: traces.

Groups of Estimated Compounds	Contents (mmol (100 g)^−1^ DW)					
	Stationary Culture		Agitated Culture		Soil-Grown Plant Raw Material (Herb)	
**(A) *Cistus* × *incanus***						
Phenolic acids	0.24b ± 0.01		0.06a ± 0.02		1.30c ± 0.02	
Catechins	0.75c ± 0.01		0.25a ± 0.02		0.50b ± 0.01	
Flavonoids	0.18b ± 0.03		0.04a ± 0.02		0.41c ± 0.02	
**(B) *Verbena officinalis***						
Phenolic acids	0.24b ± 0.03		tr		0.07a ± 0.04		
Phenylethanoid glycosides	4.53c ± 0.02		11.08b ± 0.02		1.28a ± 0.02		
**(C) *Scutellaria baicalensis* and *Scutellaria lateriflora***						
**Groups of Estimated Compounds**	**Contents (mmol (100 g)^−1^ DW)**						
	***Scutellaria baicalensis***			***Scutellaria lateriflora***			
	**Stationary Cultures**	**Soil-Grown Plant Raw Material (Root)**		**Stationary Cultures**		**Soil-Grown Plant Raw Material (Herb)**	
Phenolic acids	0.10a ± 0.02	2.16c ± 0.02		0.15a ± 0.02		0.28b ± 0.02	
Flavonoids	0.79a ± 0.04	6.26c ± 0.03		1.20b ± 0.02		1.54b ± 0.02	
Phenylethanoid glycosides	1.33c ± 0.02	0.12a ± 0.02		0.43b ± 0.02		1.15c ± 0.02	
Phenylethanoid glycosides	1.33c ± 0.02	0.12a ± 0.02		0.43b ± 0.02		1.15c ± 0.02	

## Acronyms and Symbols

ABTS	2,2’-Azino-bis(3-ethylbenzothiazoline-6-sulphonic acid)
CUPRAC	Cupric ion reducing antioxidant capacity
QUENCHER	Quick, easy, new, cheap, and reproducible treatment, involving forced solubilization of bound phenolics by oxidizing TAC (total antioxidant capacity) reagent
DPPH	1,1-diphenyl-2-picrylhydrazyl radical
DW	Dry weight
FRAP	The ferric ion reducing antioxidant potential
HPLC	High-performance liquid chromatography
MS	Murashige and Skoog
ROS	Reactive oxygen species
TAC	Total antioxidant capacity
TACA	Total antioxidant capacity assays
TEAC	Trolox equivalent antioxidant capacity
